# Multivariate logistic regression analysis on the association between anthropometric indicators of under-five children in Nigeria: NDHS 2018

**DOI:** 10.1186/s12887-021-02657-5

**Published:** 2021-04-22

**Authors:** Lijalem Melie Tesfaw, Haile Mekonnen Fenta

**Affiliations:** Department of Statistics, Bahir Dar University, Bahir Dar, Ethiopia

**Keywords:** Anthropometric indicators, Multivariate logistic regression model, Nigeria, Under-five children

## Abstract

**Background:**

Child malnutrition is a huge health problem having multifaceted consequences for child survival and long-term well-being. Although, several studies investigated stunting, underweight, and wasting in low- and middle-income countries, in Nigeria, the link between them received little attention. The aim of this study is, therefore, to assess the association between anthropometric indicators of under-five children such as stunting, underweight and wasting given that of other characteristics of children and households.

**Methods:**

The data for this study was obtained from Nigerian Demographic and health survey (NDHS) in 2018. A total of 11,314 under-five children were involved. Multivariate logistic regression model was used to determine the association between stunting, underweight and wasting given that of the estimated effect of other determinants.

**Results:**

From 11,314 under-five children the study considered 36.2, 21.4 and 6.7% of them suffered from stunting, underweight and wasting, respectively. About half (50.7%) of the children were male, 24.1% was obtained from North West region of Nigeria, and 37.8% of them were from households having unimproved drinking water. The pairwise dependency between stunting and underweight; underweight and wasting was measured using odds ratio (OR) of 15.796, and 16.750 respectively. The estimated odds of children from richest household to become stunted, underweight, and wasted was respectively 0.392, 0.540, 0.786 times that of the estimated odds of children from poorest households.

**Conclusion:**

The prevalence of under-five children with stunting, underweight and/or wasting in Nigeria was very high. The important determinants of stunting, underweight, and wasting for under five children were household wealth index, women body mass index, sex of the child, anemia, mothers’ age at first birth, and a diarrhea two weeks prior to the survey. Whereas, region, religion, multiple birth, women’s educational level significantly associated with both stunting and underweight. Both stunting and wasting significantly associated with underweight.

## Background

Child malnutrition has multifaceted consequences for child survival and long-term well-being [1]. Its prevalence has decreased globally though the decrement has not been consistent in all regions of the world. In middle- and low-income countries including sub-Saharan Africa, child malnutrition is remaining a relatively unabated challenge and still a high number of children suffer from chronic malnutrition [2]. Anthropometric indices of malnutrition such as stunting, underweight, and wasting are three indicators used to measure nutritional insufficiency or imbalance, causes of multifaceted health problem to children [3, 4]. The three are widely recognized indicators of child malnutrition status [5, 6]. More than half of the deaths in developing countries among under five associated with malnutrition. In Sub-Saharan Africa, under five children are malnourished in very high number and deaths increase on a daily basis [3].

Child malnutrition indicators stunting, wasting and underweight refers to a child: too short for his or her age (low height-for-age); too thin for his or her height (low weight-for-height); and low weight-for-age, respectively. Height-for-age, weight-for-height and weight-for-age z-scores are calculated using the 2006 WHO child growth standards. Children who have a height-forage z-score (HAZ), a weight-for-height z-score (WHZ), and weight for age z-score (WAZ) which is below two are defined as having stunting, wasting and underweight respectively [5, 7]. According to United Nations International Children’s Emergency Fund (UNICEF), environmental, economic, and socio-political factors play a major role in the malnutrition of children [4].

As a leading economy of Africa, Nigeria strives to reduce the number of its malnourished children. Yet, its children still face malnutrition. The 2013 Nigerian Demographic and Health Survey (NDHS) indicated that more than one third of its under-five had stunting [4]. The country has high fertility rate per woman associated with high rate of malnourished children. The NDHS in 2008 estimated prevalence of wasting, stunting, and underweight as being 14, 40.6, and 23.1% respectively [8]. Still, Nigeria suffers from concurrent forms of malnutrition. Globally, around 6 million children are reported to have stunting and underweight simultaneously though much of this is bunched in developing countries, especially in Africa [5].

A study in South Asia on children under 2 years indicated that stunting has significant association with poor child developmental indicators [9]. A study on Ghanaian preschool reported the prevalence of concurrent wasting and stunting among children of 0–59 months is low [3]. Studies revealed that the factors contributing to child malnutrition are multiple [5, 10, 11]. Socioeconomic inequalities, feeding practices, geographical differences, household food insecurity, and maternal literacy are among the common determinants.

A study on schoolchildren aged 7–14 years in southern Ethiopia showed that low maternal education and household food security had significant association with stunting and underweight of children [10]. Geopolitical zone (North East, North West and North Central), perceived birth size, sex of child, place/mode of delivery, and fever two weeks prior to the survey were the most consistent factors associated with under-weight and wasting [11]. A study in Pakistan reported that place of residence, Mother’s BMI, wealth index, child size at birth, mothers age at marriage and antenatal clinic visits has significant independent association with child nutritional status [5]. The study also revealed that household, in particular, wealth index has significant association with stunting, and it was higher among children from lowest socio-economic status households.

Numerous studies were done on stunting, underweight and wasting at Nigeria, Pakistan, Ethiopia, Uganda [3, 5, 12, 13]. However, little attention was given on their association and there is a paucity of literature. Though, wasting and stunting are often presented as two separate forms of malnutrition requiring different interventions for prevention and/or treatment, they are closely related and often occur together in the same populations and often in the same children. Wasting and stunting are both associated with increased mortality, especially when both are present in the same child [14]. A study conducted in Ethiopia and India [6, 7] assessed the association between the three anthropometric indicators. However, the effect of other children and household characteristics was not considered while the association is assessed. Thus, the aim of this study in Nigeria is to assess the association between anthropometric indicators of under-five children, given the other children and household characteristics. In this study, the hypotheses tested were (i) there is no relationship the three anthropometric indicators among under five children (ii) there is no relationship between socio-economic as well as demographic factors and anthropometric indicators. It is important, therefore, better understanding of the association between anthropometric indicators will help in developing focused interventions to improve child health and survival. As a result, the findings of this study will benefit policymakers at governmental and private level to provide evidence on which interventions and policy actions can be formulated and implemented for children aged under five.

## Methods

### Study design and population

A cross-sectional study design was implemented and data obtained from 11,314 under-five children in Nigeria. The data for this study was obtained from Nigerian Demographic and health survey (NDHS) of 2018 [15]. The 2018 NDHS was implemented by the National Population Commission in collaboration with the National Malaria Elimination Program of the Federal Ministry of Health, Nigeria. It is designed to provide data for monitoring the population and health situation in Nigeria. Providing reliable estimates of early childhood mortality and assessing child health problems are the main among its multidisciplinary aims.

### Inclusion/exclusion criteria

The inclusion criteria were age between below five years and completed relevant forms about the personal information and clinical signs. Hence, children not completed all relevant information or aged greater than or equal to five years were excluded.

### Sampling and data collection procedure

The sample was selected using a stratified, two-stage cluster design, to which enumeration areas (EAs) were the sampling units for the first stage. The second stage involved a complete listing of households carried out in each of the 1400 selected EAs. Based on the women’s questionnaire, in all the selected households, data of children along with its complete anthropometric indicators was considered. The information was collected as part of a retrospective birth history in which female respondents listed all of the children to whom they had given birth, along with each child date of birth, survivorship status, and current age. As the data used in this study were collected using two-level stratified cluster sampling, checking cluster effect in the dataset is relevant. The existence of clustering effect on the anthropometric indicators was checked using mean odds ratio (MOR) [16]. The MOR is defined as: MOR= $\mathit{\exp}\left\{0.6745\surd 2{\upsigma}_{\mu}^2\right\}$[16], where $\sigma \frac{2}{\mu }$ is the cluster variance. The MOR value for anthropometric indicators stunting, wasting and underweight were 0.999, 1.001, and 0.997, respectively, which are close to one. This indicates that there is no statistically significant cluster variation.

### Variables

#### Dependent variables

In this study, three dependent variables stunting, underweight, and wasting these so-called anthropometric indicators were considered. The three anthropometric indicators were measured through standardized score (z-score) for height-for-age (stunting), weight-for height (wasting) and weight-for-age (underweight). Z-score for the i^th^ child (*Z*_*i*_) is defined as: ${Z}_i=\frac{AI_i-\mu }{\sigma }$, where *AI*_*i*_, *μ*, and *σ* are anthropometric indicator of the i^th^ child, median and standard deviation, respectively. After the *Z*_*i*_ for each child is calculated, the dependent variables was recoded into binary outcomes as: stunted (0 = No if HAZ ≥ − 2 and 1 = Yes if HAZ *<* − 2), wasted (0 = No if WHZ ≥ − 2 and 1 = Yes if WHZ *< −* 2), and underweight (0 = No if WAZ ≥ − 2 and 1 = Yes if WAZ *<* − 2) according to WHO child growth standards [9].

#### Independent variables

Twenty one independent variables obtained from children and their respective households were considered (see Table 2). The selection of independent variables about factors affecting the nutritional status of children was theoretically driven [7, 12, 17]. The parameters in Table 2 collected using face to face interview of mothers.

#### Ethics approval and consent to participate

The ethical clearance for 2018 NDHS was approved by Ethical Review Board of Nigeria National Population Commission (NPC) and all participants who agreed to take part in the survey signed a consent form. Hence, author asked the permission to use data via online form and the data manager has given permission to use the data for this study.

### Statistical analysis

#### Logistic regression model

Logistic regression is a statistical model used to estimate the effect of factors when we have categorical response. In this study, let *Y*_1*i*_, *Y*_2*i*_, and *Y*_3*i*_ are binary response of stunting, underweight and wasting of the *i*^*th*^ under-five children, respectively. For binary response *Y*_*ji*_ and a vector of explanatory variables X, the logistic regression model is given by [18]:

1 $$ {\pi}_j(X)=\frac{e^{\beta_{j0}+{\beta}_{j1\chi 1}+{\beta}_{j2\chi 2}+\dots +{\beta}_{jp\chi p}}}{1+{e}^{\beta_{j0}+{\beta}_{j1\chi 1}+{\beta}_{j2\chi 2}+\dots +{\beta}_{jp\chi p}}}=\frac{e^{X{\beta}_j}}{1+{e}^{X{\beta}_j}}\kern0.5em j=1,2,3 $$

where *π*_*j*(*X*)_ = *P* (*Y*_*ji*_ = 1|*X*), the probability of the *i*^*th*^ child being stunted (*Y*_1*i*_), underweight (*Y*_2*i*_) and/or wasted (*Y*_3*i*_) given other covariates X.

Equivalently, the logit (log odds) that manifest linear relationship with explanatory variables can be expressed as:

2 $$ logit\left[{\pi}_{j(X)}\right]= logit\left[P\left({Y}_{ji}=1|X\right)\right]={\beta}_{j0}+{\beta}_{j1}{x}_1+{\beta}_{j1}{x}_1+\dots +{\beta}_{j1}{x}_1=X{\beta}_j $$

In logistic regression model the null hypothesis (*H*_0_: *β* = 0) states that the probabilities of success is independent of covariate X. The significance of each covariates is detected using wald test statistic given by *β/se*^ˆ^ (*β*^ˆ^), which has standard normal distribution for large samples. In logistic regression model the most common types of measuring association between categorical variables is odds ratio. It is the ratio of odds defined as$ORj=\frac{Oddsj1}{Oddsj2}=\frac{\pi j(X1)/\left(1-\pi j(X1)\right)}{\pi j(X2)/\left(1-\pi j(X2)\right)}$ [18].

An Odds ratio that departs or varies from 1 refers less dependency between the two categorical variables.

To perform separate analysis of stunting, underweight and wasting of under-five children as done by studies of [5–7, 11, 12], applying ordinary logistic regression is sufficient. However, doing so would ignore the dependency between the three anthropometric indicators. To address this, to take into account the correlation between the anthropometric indicators and the estimates of effects of other covariates, multivariate logistic regression is more plausible alternative. This statistical model serves to model two or more than two categorical outcomes of interests at a time and assess their association given that of other covariates [19]. It enables to model the marginal probabilities as a function of a set of child and household characteristics, and at the same time account for the correlation among the three anthropometric indicators.

The data was initially imported and organized using SPSS software. Finally the analysis was carried out using R of package VGAM [20]. VGAM was used to provide functions for fitting vector generalized linear and additive models.

#### Goodness of fit test

Prior to fitting the model, checking the adequacy or goodness of fit of the model is needed. This could be detected using the predictive power of the estimated model. In logistic regression model, predictive power is commonly measured or detected using the concordance index. Concordance index estimates the probability that the predictions and the outcomes are concordant, which is whether that the estimated outcome matched with the observed outcome [18]. Thus, in this study, concordance index was computed to check how well the estimated model fits the data.

## Results

From a total of 11,314 under-five children considered in this study 36.2, 21.4 and 6.7% of children suffered from stunting, underweight and wasting respectively (see Table 1).

**Table 1:** Dependent variables description and frequency distribution

Variables	Categories (codes)	n(%)
Stunting	no (0)	7213(63.8)
	yes (1)	4101(36.2)
underweight	no (0)	8890(78.6)
	yes (1)	2424(21.4)
wasting	no (0)	10,556(93.3)
	yes (1)	758(6.7)

More than 20 % (20.8%) of the children were aged between 48 and 59 months, almost half of children (50.7%) were male, and 60.8% of these children live in rural. The majority of children (24.1%) was obtained from North West region of Nigeria while 37.8% of them were from households having unimproved drinking water. Most of the households (41.9%) have two under-five children. Mothers and husbands who did not attend any formal education were 37.8 and 28.8% respectively. More than half of the children (54.1%) were still being breastfed. The highest proportion of children (26.0%) was from Hausa Ethnicity, while the lowest (0.5%) was obtained from Ekoi ethnic group. Two weeks prior to the survey, 17.0, 26.0, 12.7, and 28.4% of children had cough, fever, diarrhea, and anemia respectively (see Table 2).

**Table 2:** Independent variables description and frequency distribution

Variables	Categories (codes)	n (%)
Age of child	0–5(0)	1114(9.8)
	6–11(1)	1163(10.3)
	12–23(2)	2235(19.8)
	24–35(3)	2187(19.3)
	36–47(4)	2261(20.0)
	48–59(5)	2354(20.8)
Sex of child	male (0)	5732(50.7)
	female (1)	5582(49.3)
Number of under five children in the household	1(0)	3042(26.9)
	2(1)	4744(41.9)
	3 and more (2)	3528(31.2)
Residence	Urban (0)	4431(39.2)
	Rural (1)	6883(60.8)
Region	North central (0)	1993(17.6)
	North East (1)	2005(17.7)
	North West (2)	2725(24.1)
	South East (3)	1661(14.7)
	South South (4)	1265(11.2)
	South West (5)	1665(14.7)
Household size	Small (1–4)(0)	2874(25.4)
	medium(5–9)(1)	6348(56.1)
	Large (10 and more) (2)	2092(18.5)
Source of drinking water	Unimproved (0)	4272(37.8)
	improved (1)	7042(62.2)
Breast feeding	No (0)	5190(45.9)
	Yes (1)	6124(54.1)
Birth oder	1st (0)	2177(19.2)
	2–3(1)	3890(34.4)
	4–5(2)	2730(24.1)
	6 and more (3)	2517(22.2)
Multiple birth Cough	single birth (0)	10,958(96.9)
	1st of multiple (1)	198(1.8)
	2nd of multiple (2)	158(1.4)
	No (0)	9389(83.0)
	Yes (1)	1925(17.0)
Fever	No (0)	8376(74.0)
	Yes (1)	2938(26.0)
Diarrhea	No (0)	9882(87.3)
	Yes (1)	1432(12.7)
Anemia	Not anemic (0)	3217(28.4)
	Anemic (1)	6922(61.2)
	Missing	1175(10.4)
Religion	Catholic (0)	1169(10.3)
	Other Christian (1)	4136(36.6)
	Islam (2)	5908(52.2)
	Traditionalist and others (3)	101(0.9)
Mothers’ Age at 1st birth	*>* 20(0)	8000(70.7)
	20–34(1)	3268(28.9)
Mothers’Education level	35–49(2)	46(0.4)
	No education (0)	4276(37.8)
	Primary (1)	1913(16.9)
	Secondary and higher (2)	5125(45.3)
Husbands’Education level	No education (0)	3256(28.8)
	Primary (1)	1605(14.2)
	Secondary and higher (2)	5892(52.1)
	Missing	561(5.0)
Wealth index	poorest (0)	2225(19.7)
	poorer (1)	2258(20.0)
	middle (2)	2502(22.1)
	richer (3)	2402(21.2)
	richest (4)	1927(17.0)
Body mass index	Thin (0)	1068(9.4)
	medium (1)	7065(62.4)
	overweight (2)	3102(27.4)
	missing	79(0.7)
Ethnicity	Ekoi(0)	58(0.5)
	Fulani(1)	891(7.9)
	Hausa(2)	2946(26.0)
	Ibibio(3)	201(1.8)
	Igala(4)	112(1.0)
	Igbo(5)	1923(5)
	Ijaw/Izon(6)	336(3.0)
	Kanuri/Beriberi(7)	241(2.1)
	Tiv (8)	276(2.4)
	Yoruba (9)	1444(12.8)
	Other (10)	2886(25.5)

The result in Table 1 shows a child victimized by only one of the three child malnutrition. In this sense, a child experiences stunting, underweight, and wasting in mutually exclusive manner. Tables 3 and 4, however, indicated that children were infected by more than one of the three children malnutrition, and hence caution is needed in interpreting the total malnourished children. For instance, 1704 children had both stunting and underweight. For the sake of simplicity, the information in Table 3 could be as in Table 4.

**Table 3:** Concurrent frequency distribution of stunting, underweight and wasting

				Stunting		Total
				No	Yes	
Underweight	No	Wasted	No	6676	2048 0	8724
			Yes	166	1704	166
	Yes	Wasted	No	128	349	1832
			Yes	243		592
Total				7213	4101	11,314

**Table 4:** Cross classification of malnourished category and corresponding frequency distribution

malnurished category	frequency (%)
non-malnurished	6676 (59.0)
stunting only	2048 (18.1)
Wasting only	166 (1.5)
Underweight only	128 (1.1)
Stunting and Underweight	1704 (15.1)
Underweight and Wasting	243 (2.1)
Stunting, Wasting, and Underweight	349 (3.1)

Table 4 shows the prevalence of children with their corresponding maturational status (anthropometric indices) along with the frequency computed from Table 3. In Table 4 the sample children were categorized into eight non-overlapping categories as: non-malnourished (a child with none of the three anthropometric indices or a child is nourished), stunting only, underweight only, wasting only, stunting and underweight, stunting and wasting, underweight and wasting, and finally a child with stunting, underweight, and wasting. The prevalence of proportion of children with a stunting only was (18.1%), the highest compared to other categories of malnourishment. Children with stunting and underweight (15.1%) ranked as the second highest. In total 41.0% (={18.1+ 1.5 + 1.1 + 15.1 + 2.1 + 3.1}%) of the children were malnourished.

Table 5 shows all possible pairwise dependency between the three anthropometric indicators of stunting, underweight, and wasting using odds ratio (OR). The odds ratio for the dependency between stunting and underweight; stunting and wasting; and underweight and wasting were 18.487, 1.547, and 16.980, respectively. This differed differ from unity. This indicates a dependency between the three anthropometric indicators, and hence fitting a multivariate logistic model for the three responses is appropriate to incorporate their dependency and estimate the effects of the covariates. Therefore, the multivariate logistic regression analysis of stunting, underweight and wasting given that of other covariates was presented in Table 7.

**Table 5:** Measuring possible pairwise dependency between stunting, underweight and wasting using OR

	Underweight	Wasting
Stunting	18.487	1.547
Underweight		16.980

Table 6 revealed the bivariate analysis on the association between covariates of each anthropometric indicator and the distribution of under five in each levels of the covariate. Sex of child, number of under five children in the household, residence, region, source of drinking water, breast feeding, birth order, fever, diarrhea, anemia, religion, mother’s and husband’s education level, mothers’ body mass index, wealth index, and ethnicity were covariates independently associated (*p*-value*<* 0.05) with stunted, underweight and wasted.

**Table 6:** Bivariate analysis on the association between covariates and each anthropometric indicator

Covariates	Stunting		p-value	Underweight		p-value	Wasting		p-value
	Yes(%)	No(%)		Yes(%)	No(%)		Yes(%)	No(%)	
Age of child									
0–5	405(9.9)	709(9.8)		251(10.4)	863(9.7)		74(9.8)	1040(9.9)	
6–11	418(10.2)	745(10.3)		268(11.1)	895(10.1)		80(10.6)	1083(10.3)	
12–23	789(19.2)	1446(20.0)		468(19.3)	1767(19.9)		156(20.6)	2079(19.7)	
24–35	808(19.7)	1379(19.1)	0.920	470(19.4)	1717(19.3)	0.467	155(20.4)	2032(20.1)	0.820
36–47	823(20.1)	1438(19.9)		488(20.1)	1773(19.9)		138(18.2)	2123(20.1)	
48–59	858(20.9)	1496(20.7)		479(19.8)	1875(21.1)		155(20.4)	2199(20.8)	
Sex of child male female	2220(54.1)	3512(48.7)		1293(53.3)	4439(49.9)		448(59.1)	5284(50.1)	
	1881(45.9)	3701(51.3)	0.000	1131(46.7)	4451(50.1)	0.002	310(40.9)	5272(49.9)	0.000
No. of children(*<* 5 *yrs*) one two	949(23.1)	2093(29.0)	0.000	559(23.1)	2483(27.9)	0.000	182(24.0)	2860(27.1)	0.008
	1641(40.0)	3103(43.0)		949(39.2)	3795(42.7)		302(39.8)	4442(42.1)	
three and more	1511(36.8)	2017(28.0)		916(37.8)	2612(29.4)		274(36.1)	3254(30.8)	
Residence									
Urban	1154(28.1)	3277(45.4)		657(27.1)	3774(42.5)		228(30.1)	4203(39.8)	
Rural	2947(71.9)	3936(54.6)	0.000	1767(72.9)	5116(57.6)	0.000	530(69.9)	6353(60.2)	0.000
Region									
North central	566(13.8)	1427(19.8)		294(12.1)	1699(19.1)		106(14.0)	1887(17.9)	
North East	976(23.8)	1029(14.3)		585(24.1)	1420(16.0)		188(24.8)	1817(17.2)	
North West	1575(38.4)	1150(15.9)		984(40.6)	1741(19.6)		257(33.9)	2468(23.4)	
South East	319(7.8)	1342(18.6)	0.000	187(7.7)	1474(16.6)	0.000	81(10.7)	1580(15.0)	0.000
South South	282(6.9)	983(13.6)		149(6.1)	1116(12.6)		52(6.9)	1213(11.5)	
South West	383(9.3)	1282(17.8)		225(9.3)	1440(16.2)		74(9.8)	1591(15.1)	
Household size small (1–4) medium(5–9)	848(20.7)	2026(28.1)		534(22.0)	2340(26.3)		187(24.7)	2687(25.5)	
	2187(53.3)	4161(57.7)	0.000	5089(57.2)	1259(51.9)	0.000	410(54.1)	5938(56.3)	0.130
large(10 and more)	1066(26.0)	1026(14.2)		631(26.0)	1461(16.4)		161(21.2)	1931(18.3)	
Source of drinking water	1860(45.4)	2412(33.4)	0.000	1089(44.9)	3183(35.8)	0.000	321(42.3)	3951(37.4)	0.004
unimproved improved	2241(54.6)	4801(66.6)		1335(55.1)	5707(64.2)		437(57.7)	6605(62.6)	
Breast feeding									
No	1934(47.2)	3256(45.1)		1037(42.8)	4153(46.7)		256(33.8)	4934(46.7)	
Yes	2167(52.8)	3957(54.9)	0.020	1387(57.2)	4737(53.3)	0.000	502(66.2)	5622(53.3)	0.000
Birth order									
1st	650(15.8)	1527(21.2)		383(15.8)	1794(20.2)		131(17.3)	2046(19.4)	
2–3	1249(30.5)	2641(36.6)		741(30.6)	3149(35.4)		236(31.1)	3654(34.6)	
4–5	1023(24.9)	1707(23.7)	0.000	563(23.2)	2167(24.4)	0.000	174(23.0)	2556(24.2)	0.000
6 and more	1179(28.7)	1338(18.5)		737(30.4)	1780(20.0)		217(28.6)	2300(21.8)	
Multiple birth									
Single birth	3926(95.7)	7032(97.5)		2310(95.3)	8648(97.3)		726(95.8)	10,232(96.9)	
1st of multiple	94(2.3)	104(1.4)	0.000	57(2.4)	141(1.6)	0.000	16(2.1)	182(1.7)	0.160
2nd of multiple	81(2.0)	77(1.1)		57(2.4)	101(1.1)		16(2.1)	142(1.3)	
Cough									
No	3422(83.4)	5967(82.7)		2009(82.9)	7380(83.0)		608(80.2)	8781(83.2)	
Yes	679(16.6)	1246(17.3)	0.329	415(17.1)	1510(17.0)	0.875	150(19.8)	1775(16.8)	0.035
Fever									
No	2851(69.5)	5525(76.6)		1620(66.8)	6756(76.0)		485(64.0)	7891(74.8)	
Yes	1250(30.5)	1688(23.4)	0.000	804(33.2)	2134(24.0)	0.000	273(36.0)	2665(25.2)	0.000
Diarrhea									
No	3399(82.9)	6483(89.9)		1944(80.2)	7938(89.3)		601(79.3)	9281(87.9)	
Yes	702(17.1)	730(10.1)	0.000	480(19.8)	952(10.7)	0.000	157(20.7)	1275(12.1)	0.000
Anemia Not anemic	974(25.0)	2243(35.9)	0.000	502(22.4)	2715(34.4)	0.000	144(21.2)	3073(32.5)	0.000
Anemic	2916(75.0)	4006(64.1)		1735(77.6)	5187(65.6)		535(78.8)	6387(67.5)	
Religion									
Catholic	234(5.7)	935(13.0)		129(5.3)	1040(11.7)		55(7.3)	1114(10.6)	
Other Christian	967(23.6)	3169(43.9)		519(21.4)	3617(40.7)		192(25.3)	3944(37.4)	
Islam	2871(70.0)	3037(42.1)	0.000	1762(72.7)	4146(46.6)	0.000	508(67.0)	5400(51.2)	0.000
Traditionalist and others	29(0.7)	72(1.0)		14(0.6)	87(1.0)		3(0.4)	98(0.9)	
Mothers’ Age at 1st birth									
*<* 20	2931(71.5)	5069(70.3)		1582(65.3)	6418(42.2)		460(60.7)	7540(71.4)	
20–34	1158(28.2)	2110(29.3)	0.172	833(34.4)	2435(27.4)	0.000	295(38.9)	2973(28.2)	0.000
35–49	12(0.3)	34(0.5)		9(0.4)	37(0.4)		3(0.4)	43(0.4)	
Mothers’ education level									
No education	2302(56.1)	1974(27.4)		1461(60.3)	2815(31.7)		412(54.4)	3864(36.6)	
Primary	710(17.3)	1203(16.7)	0.000	344(14.2)	1569(17.6)	0.000	101(13.3)	1812(17.2)	0.000
Secondary and higher	1089(26.6)	4036(56.0)		619(25.5)	4506(50.7)		245(32.3)	4880(46.2)	
Husbands’ education level									
No education	1746(44.3)	1510(22.2)		1107(47.3)	2149(25.5)		321(44.0)	2935(29.3)	
Primary	626(15.9)	979(14.4)	0.000	338(14.5)	1267(15.1)	0.000	95(13.0)	1510(15.1)	0.000
Secondary and higher	1567(39.8)	4325(63.5)		893(38.2)	4999(59.4)		314(43.0)	5578(55.7)	
Mothers’ Body mass index									
Thin	527(13.0)	541(7.5)		421(17.5)	647(7.3)		154(20.4)	914(8.7)	
Medium	2816(69.3)	4249(59.2)	0.000	1625(67.7)	5440(61.6)	0.000	482(63.8)	6583(62.8)	0.000
Overweight	720(17.7)	2382(33.2)		353(14.7)	2749(31.1)		119(15.8)	2983(28.5)	
Wealth index poorest poorer middle	1229(30.0)	996(13.8)	0.000	786(32.4)	1439(16.2)	0.000	233(30.7)	1992(18.9)	
	1065(26.0)	1193(16.5)		612(25.2)	1646(18.5)		168(22.2)	2090(19.8)	
	910(22.2)	1592(22.1)		506(20.9)	1996(22.5)		162(21.4)	2340(22.2)	0.000
richer	594(14.5)	1808(25.1)		332(13.7)	2070(23.3)		118(15.6)	2284(21.6)	
richest	303(7.4)	1624(22.5)		188(7.8)	1739(19.6)		77(10.2)	1850(17.5)	
Ethnicity									
Ekoi	14(0.3)	44(0.6)		8(0.3)	50(0.6)		4(0.5)	54(0.5)	
Fulani	462(11.3)	429(5.9)		316(13.0)	575(6.5)		81(10.7)	810(7.7)	
Hausa	1623(39.6)	1323(18.3)		1004(41.4)	1942(21.8)		284(37.5)	2662(25.2)	
Ibibo	42(1.0)	159(2.2)		25(1.0)	176(2.0)		10(1.3)	191(1.8)	
Igala	26(0.6)	86(1.2)		5(0.2)	107(1.2)		2(0.3)	110(1.0)	
Igbo	341(8.3)	1582(21.9)	0.000	210(8.7)	1713(19.3)	0.000	94(12.4)	1829(17.3)	0.000
Ijaw/Izon	82(2.2)	254(3.5)		44(1.8)	292(3.3)		8(1.1)	328(3.1)	
Kanuri/Beriberi	126(3.1)	115(1.6)		95(3.9)	146(1.6)		48(6.3)	193(1.8)	
Tiv	60(1.5)	216(3.0)		29(1.2)	247(2.8)		18(2.4)	258(2.4)	
Yoruba	351(8.6)	1093(15.2)		204(8.4)	1240(13.9)		64(8.4)	1380(13.1)	
Others	974(23.8)	1912(26.5)		484(20.0)	2402(27.0)		145(19.1)	2741(26.0)	

The proportion of stunted, underweight, and wasted among male was 54.1, 53.3, and 59.1% respectively, a figure that is higher that of than females. Compared to the proportion of children from urban area, rural the proportion of children from had stunted (71.9%), underweight (72.9%), and wasted (69.9%) were higher. The prevalence of stunted, underweight, and wasted in North West region was 38.4, 40.6, and 33.9% respectively which was higher compared to other regions in Nigeria.

Children whose mothers did not attend any formal education were more likely to be stunted (56.1%), underweight (60.3%), and wasting (54.4%). The same was true for husbands’ education level. When household wealth index declined from richest to poorest, the proportion of stunted, underweight, and wasted children increased from 7.4 to 30.0%, 7.8 to 32.4%, and 10.2 to 30.7%, respectively. The children with the highest percentage of stunted (39.6%), underweight (41.4%), and wasted (37.5%) were from Hausa ethnicity. Children from mothers’ whose age was less than 20 were more likely to be stunted (71.5%), underweight (65.3%), and wasted (60.7%) compared to children from mothers’ age 20 years and above.

Table 7 revealed the estimated effect of covariates on stunting, underweight and wasting by taking into account the dependency of stunting, underweight and wasting using multivariate logistic regression model. The pairwise dependency between stunting and underweight; stunting and wasting; underweight and wasting given other covariates were measured using OR was 15.796, 1.132, and 16.750, respectively. A value of OR that differs from one indicates a significant dependency. The OR for the dependency between stunting and underweight as well as underweight and wasting deviates significantly from one indicates a significant dependency. The OR for the dependency between stunting and wasting was 1.132 which is closer to one indicates insignificant dependency (*p*-value = 0.1767 *>* 0.05). After the possible pairwise dependency between child stunting, underweight and wasting was checked, the effect of each covariate on stunting, underweight, and wasting was estimated.

**Table 7:** Parameter estimation using multivariate logistic regression model of stunting, underweight, and wasting

Variables	Stunting		Underweight			Wasting
	est.(se)	OR (95% CI)	est.(se)	OR (95% CI)	est.(se)	OR (95% CI)
Intercept	0.433(0.433)	0.648(0.277,1.516)	0.604(0..474)	0.546(0.216,1.382)	_1.699(0.662)	0.183(0.050,0.669)
Region						
North Central(^a^)						
North East	0.560(0.085)	1.750(1.481,2.069)	0.340(0.101)	1.405(1.154,1.711)	0.108(0.162)	1.114(0.812,1.530)
North West	0.909(0.095)	2.482(2.060,2.991)	0.644(0.109)	1.904(1.539,2.355)	0.068(0.173)	1.070(0.763,1.501)
South East	0.377(0.198)	1.459(0.989,2.151)	0.215(0.239)	1.239(0.776,1.980)	_0.073(0.341)	0.930(0.476,1.815)
South South	0.068(0.128)	1.071(0.833,1.376)	0.114(0.165)	1.121(0.811,1.550)	0.095(0.257)	1.100(0.664,1.821)
South West	0.075(0.124)	1.078(0.845,1.376)	0.104(0.155)	1.109(0.818,1.504)	_0.131(0.259)	0.878(0.528,1.458)
Residence						
Urban(^a^)						
Rural	0.105(0.060)	1.111(0.988,1.248)	0.164(0.069)	1.178(1.029,1.350)	0.093(0.111)	1.099(0.883,1.365)
Religion						
Catholic(^a^)						
Other Christian	0.098(0.100)	1.103(0.906,1.344)	0.197(0.127)	1.218(0.949,1.563)	0.022(0.188)	1.022(0.706,1.478)
Islam	0.296(0.121)	1.345(1.061,1.704)	0.350(0.151)	1.419(1.054,1.911)	0.057(0.240)	1.058(0.661,1.693)
Others	0.189(0.283)	0.828(0.475,1.441)	0.053(0.356)	0.949(0.472,1.908)	0.001(0.323)	1.001(0.295,3.391)
Wealth Index						
Poorest(^a^)						
Poorer	0.100(0.073)	0.905(0.784,1.043)	0.163(0.076)	0.849(0.731,0.986)	_0.282(0.120)	0.754(0.596,0.955)
Middle	0.270(0.080)	0.763(0.652,0.893)	0.252(0.088)	0.778(0.655,0.923)	_0.193(0.139)	0.825(0.629,1.082)
Richer	0.583(0.094)	0.558(0.465,0.670)	0.489(0.107)	0.613(0.497,0.756)	_0.386(0.172)	0.680(0.486,0.952)
Richest	0.937(0.115)	0.392(0.313,0.491)	0.617(0.134)	0.540(0.415,0.701)	_0.240(0.208)	0.786(0.523,1.182)
Multiple Birth						
Single birth(^a^)						
1st of multiple	0.445(0.172)	1.560(1.113,2.186)	0.321(0.193)	1.380(0.944,2.015)	0.317(0.297)	1.373(0.766,2.459)
2nd of multiple	0.652(0.194)	1.919(1.312,2.806)	0.756(0.204)	2.131(1.428,3.179)	0.358(0.331)	1.431(0.747,2.738)
ever						
No(^a^)						
Yes	0.027(0.058)	0.973(0.869,1.089)	0.107(0.062)	1.113(0.985,1.257)	0.243(0.096)	1.275(1.055,1.539)
Child Age						
0–5(^a^)						
6–11	0.022(0.104)	0.979(0.799,1.199)	0.120(0.114)	1.127(0.902,1.409)	0.178(0.182)	1.195(0.837,1.706)
12–33	0.041(0.090)	0.959(0.803,1.145)	0.040(0.101)	0.961(0.788,1.171)	0.102(0.162)	1.107(0.806,1.520)
24–35	0.007(0.090)	1.008(0.844,1.203)	0.048(0.101)	0.953(0.782,1.162)	0.184(0.160)	1.202(0.878,1.645)
36–47	0.027(0.090)	1.028(0.861,1.227)	0.020(0.101)	0.981(0.804,1.195)	_0.039(0.164)	0.962(0.697,1.328)
48–59	0.034(0.090)	1.034(0.867,1.232)	0.051(0.101)	0.951(0.781,1.158)	0.100(0.161)	1.105(0.806,1.514)
Education levels of women						
No education(^a^)						
Primary	0.0198(0.077)	1.020(0.877,1.186)	0.223(0.088)	0.800(0.672,0.951)	_0.123(0.148)	0.885(0.662,1.183)
Secondary and above	0.300(0.081)	0.741(0.632,0.868)	0.316(0.096)	0.729(0.604,0.880)	0.010(0.154)	1.010(0.747,1.366)
Ethnicity						
Ekoi(^a^)						
Fulani	0.245(0.410)	0.783(0.351,1.748)	0.638(0.445)	0.528(0.221,1.264)	_0.788(0.614)	0.455(0.136,1.516)
Hausa	0.085(0.407)	0.919(0.414,2.042)	0.619(0.443)	0.539(0.226,1.283)	_0.518(0.609)	0.595(0.180,1.965)
Ibibo	0.437(0.434)	0.646(0.276,1.510)	0.769(0.476)	0.463(0.182,1.179)	_0.810(0.662)	0.445(0.122,1.628)
Igala	0.274(0.468)	0.760(0.304,1.901)	2.045(0.680)	0.129(0.034,0.490)	_2.276(1.170)	0.103(0.010,1.018)
Igbo:1	0.652(0.428)	0.521(0.225,1.204)	0.704(0.466)	0.495(0.200,1.233)	_0.584(0.631)	0.558(0.162,1.923)
Ijaw/Izon	0.094(0.411)	0.910(0.407,2.038)	0.710(0.454)	0.492(0.202,1.196)	_1.728(0.712)	0.178(0.044,0.718)
Kanuri/Beriberi	0.165(0.429)	0.848(0.366,1.964)	0.296(0.463)	0.744(0.300,1.843)	_0.290(0.629)	1.336(0.390,4.581)
Tiv	0.545(0.430)	0.580(0.249,1.347)	1.106(0.486)	0.331(0.128,0.857)	_0.577(0.653)	0.562(0.156,2.019)
Yoruba	0.017(0.415)	1.017(0.451,2.293)	0.510(0.455)	0.600(0.246,1.464)	_0.873(0.636)	0.4178(0.120,1.453)
Others	−0.166 (0.397)	0.849(0.390,1.844)	− 0.890(0.431)	0.411(0.176,0.956)	_.034(0.588)	0.356(0.112,1.126)
Body mass index of women						
Thin(^a^)						
Medium	0.106(0.078)	0.900(0.773,1.047)	0.501(0.079)	0.606(0.519,0.707)	_.0.555(0.111)	0.574(0.462,0.713)
Overweight	0.459(0.091)	0.632(0.529,0.755)	0.946(0.100)	0.388(0.319,0.472)	_1.011(0.155)	0.365(0.270,0.495)
Sex of child						
Male(^a^)						
Female	0.220(0.047)	0.802(0.732,0.879)	0.143(0.052)	0.867(0.782,0.961)	_.0.394(0.084)	0.675(0.572,0.796)
Cough						
No(^a^)						
Yes	0.054(0.067)	0.947(0.830,1.080)	0.020(0.075)	0.980(0.847,1.135)	_.0.049(0.114)	1.050(0.840,1.314)
Source of drinking water.						
Unimproved(^a^)						
Improved	0.032(0.053)	0.968(0.872,1.073)	0.012(0.059)	0.988(0.881,1.108)	0.036(0.093)	1.037(0.864,1.244)
Household Size						
Small (1–4)(^a^)						
Medium (5–9)	0.055(0.069)	1.056(0.922,1.210)	0.153(0.800)	0.859(0.733,1.005)	0.225(0.127)	0.799(0.622,1.025)
Large (10 and more):1	0.227(0.097)	1.255(1.038,1.518)	0.108(0.109)	0.898(0.725,1.112)	0.353(0.176)	0.702(0.498,0.991)
Education levels of household						
No education(^a^)						
Primary	0.059(0.081)	1.061(0.905,1.243)	0.083(0.089)	0.920(0.773,1.096)	0.222(0.146)	0.801(0.602,1.066)
Secondary and above	0.055(0.072)	0.946(0.822,1.089)	0.065(0.079)	0.938(0.803,1.095)	0.093(0.128)	0.911(0.710,1.170)
Breast Feeding						
No(^a^)						
Yes	0.274(0.050)	0.760(0.689,0.839)	0.025(0.056)	0.976(0.874,1.088)	0.362(0.090)	1.436(1.203,1.714)
Anemia						
No(^a^)						
Yes	0.324(0.053)	1.387(1.247,1.533)	0.329(0.062)	1.389(1.231,1.568)	0.328(0.102)	1.389(1.137,1.696)
Number of under five						
One(^a^)						
Two	0.104(0.062)	1.109(0.981,1.254)	0.008(0.072)	1.008(0.874,1.162)	_0.061(0.116)	0.941(0.750,1.181)
Three and more	0.184(0.079)	1.202(1.030,1.402)	0.133(0.089)	1.142(0.959,1.360)	0.096(0.141)	1.101(0.834,1.452)
Birth order						
1st(^a^)						
2–3	0.004(0.074)	1.004(0.868,1.160)	0.062(0.085)	1.064(0.900,1.258)	_0.068(0.132)	0.935(0.721,1.211)
4–5	0.063(0.083)	1.065(0.905,1.252)	0.067(0.096)	1.069(0.886,1.290)	0.022(0.150)	1.023(0.762,1.373)
6 and more	0.030(0.087)	1.031(0.869,1.223)	0.219(0.098)	1.245(1.026,1.509)	0.191(0.154)	1.210(0.895,1.637)
Diarrhea						
No(^a^)						
Yes	0.258(0.070)	1.295(1.129,1.484)	0.413(0.073)	1.512(1.311,1.744)	0.364(0.108)	1.439(1.164,1.780)
Mothers’ Age at first birth						
*<* 20(^a^)						
20–34	0.148(0.052)	0.863(0.779,0.955)	0.280(0.057)	1.324(1.185,1.479)	0.411(0.086)	1.509(1.275,1.786)
35–49	0.868(0.419)	0.420(0.184,0.955)	0.346(0.471)	0.708(0.281,1.783)	0.616(0.582)	1.852(0.592,5.795)
**Dependency**	Odds Ratio (OR)		P-value			
Stunting/Underweight	15.796		0.0001			
Stunting/Wasting	1.132		0.1767			
Underweight/wasting	16.750		0.0001			

Key: ^a^; Reference group *OR* Odds Ratio, *CI* Confidence Interval

The covariates in the study include household wealth index, women body mass index, sex of child, anemia, diarrhea and mothers’ age at first birth all found to be common determinants that significantly associated with stunting, underweight and wasting. Besides, region and religion of the mothers, multiple birth and education levels of women significantly associated with stunting and underweight. Household size and current breast-feeding status significantly associated with stunting and wasting. Ethnicity was the only covariate that associated with both underweight and wasting. Moreover, number of under five children in the household, place of residence, and fever were covariates that significantly associated with stunting, underweight and wasting, respectively.

The estimated odds of a child from a richest household to be stunted, underweight, and wasted was 0.392, 0.540, and 0.786 times the estimated odds of a child from a poorest household, respectively. This indicates that a child from poorest household is more likely have stunting, underweight and wasting compared to a child from a richest household. The same was true when a child from poorest household compared with poorer, middle, and richer household. The estimated odds of a female child to be stunting, underweight, and wasting was 0.802, 0.867, and 0.675 times the estimated odds of a male child, respectively. This refers to the estimated odds of a female child to be stunted, underweight, and wasted was lower by 19.8, 13.3, and 32.5% of the estimated odds of a male child. On the other hand, a female child was less likely to be stunted, underweight and wasted compared to a male child. The estimated odds of anemic children to be stunted, underweight, and wasting was 1.387, 1.389, and 1.389 times the estimated odds of non-anemic children, respectively which suggests that anemic children had higher risk of stunting, underweight, and wasted compared to non-anemic children. Compared to children from households who were following catholic religion, children from Christian household and from Islam household had higher risk of stunting and underweight.

The risk of children to be stunted and underweight was higher in North East, North West, South East, South South, and South West compared with North Central region of Nigeria. For instance, the estimated odds of children from North West to be stunted and underweight was 2.482 and 1.904 times the estimated odds of children from North Central, respectively. The estimated odds of children whose mothers attended secondary and above education to be stunted and underweight were 0.741 and 0.729 times the estimated odds of children whose mothers had never attended formal education, respectively. As the education level of mothers getting increased, the probability that a child becomes stunted and underweight is lower. There was also significant underweight and wasting difference of children based on ethnicity. The estimated odds of children from Ibibo to be underweight was 0.129 times the estimated odds of children from Ekoi ethnicity. Besides this, the estimated odds of children from Kanuri/Beriberi to be wasting was 1.336 times the estimated odds of children from Ekoi ethnicity. This corresponds with children from Kanuri/Beriberi ethnicity were more likely to be wasting compared to children from Ekoi ethnicity.

Based on the estimated multivariate logistic regression model of stunting, underweight and wasting the proportion of concordant and dis-concordant was computed in Table 8. Concordant value of 78.9% shows that as many as 78.9% of children with anthropometric indices such as stunting, underweight and/or wasted would have better chance in predicting the category stunting, underweight and/or wasted. In this regard, the concordant index is very high which indicates that the adequacy of the model adequacy in reflecting the association was very good and fits the data well.

**Table 8:** Goodness of fit test using concordant and dis-concordant proportion

	Concordant	Dis-concordant
Proportion	78.9%	21.1%

## Discussion

In this study, the association between anthropometric indices such as stunting, underweight, and wasting of under-five children given other covariates/characteristics was briefly discussed and the effects of covariates were estimated. The anthropometric indicators height-for-age standardized score (HAZ), weight-for-age standardized score (WAZ), and weight-for-height standardized score (WHZ) were recoded into binary outcomes of stunting, underweight, and wasting respectively [7]. Multivariate logistic regression model was employed to determine the effects of covariates on stunting, underweight and wasting. Based on this model, significant dependencies between stunting and underweight, and underweight and wasting were noticed given other children and household characteristics. The findings of this study was consistent with studies conducted in Ethiopia and India [6, 7] which reported that underweight significantly associated with both stunting and wasting. These studies also revealed that underweight is a composite measure of stunting and wasting. Nevertheless, in these studies, when the association between the three anthropometric indicators was assessed, the effect of other children and households’ characteristics was not taken into consideration. On the other hand, there was insignificant association between stunting and wasting which is in line with a study on Ghanaian preschool children [2] which suggested that the prevalence of concurrent stunting and wasting among under-five children was low.

The prevalence of child malnutrition in Nigeria (41%) was very high, though varied geographically. In North West Nigeria the proportion stunting, underweight, and wasting was 38.4, 40.6, and 33.9% which compared to other regions was very high. This was in line with a study by Duru et al. [4],which reported that the prevalence of child malnutrition varied significantly not only among different nations of the world but also in different regions of a country. Compared with North central region of Nigeria, the risk of children to be stunted and underweight was higher in North East, North West, South East, South South, and South West. Similarly, compared to urban children, rural children were more likely to be underweight. This was consistent with study results from at Kwara State of Nigeria [3], where limited infrastructure such as health care facilities, inadequate food, and nutrient supply led to high rate of rural malnutrition.

Household wealth index, women body mass index, sex of child, anemia, diarrhea two weeks prior to the survey, and mothers’ age at first birth were important determinants of stunting, underweight, and wasting. Consistent with study findings from studies in Pakistan and Ethiopia [5, 13] which associated household wealth index with child malnutrition, children from poorest households were more likely to be malnourished. Children from households of low wealth index experienced challenges of household food security, health care basic necessity which impact the growth of children. Unlike studies in Pakistan [5], Uganda and Nigeria [11, 12], females children from Nigeria were vulnerable to stunting, stunting, underweight and wasting more significantly than males children. Females children were less likely to be stunted, underweight, and wasted compared to males. As the level of maternal education increased, the probability of children to be stunted and underweight was lower, which was consistent with study findings in Ethiopia [21].

Thus, given the high-rate child malnutrition, the researchers suggest policy makers and stakeholders reduce the burden through multidimensional interventions. To overcome the shortcomings of this cross-sectional study, future studies should employ methods that allow inferring causality so as to be able to see associations between child anthropometric indicators given that of other characteristics.

## Conclusion

The prevalence of under-five children with stunting, underweight and/or wasting in Nigeria was very high. The study found significant association between both stunting and wasting and underweight. On the other side, no association was noticed between stunting and wasting. Household wealth index, women body mass index, sex of child, anemia, diarrhea two weeks prior to the survey, and mothers’ age at first birth were found important determinants of stunting, underweight, and wasting of under five children. Finally, region, religion, multiple birth and educational level of women significantly associated with both stunting and underweight.

## Data Availability

The datasets for generated analyses during the study is available in Nigeria Demographic and. Health Survey data if unique request sent via their site NDHS 2018.

## Abbreviations

NDHS: Nigerian Demographic and Health Survey
HAZ: Height for age standardized score
WAZ: Weight for age standardized score
WHZ: Weight for height standardized score
OR: Odds ratio
UNICEF: United Nations International Children’s Emergency Fund

## References

[1] UNICEF: Nigeria Nutrition profile Nigeria-Nutrition-Profile-Mar2018. Retrieved oct. 2020.

[2] Saaka M, Galaa SZ (2016). Relationships between wasting and stunting and their concurrent occurrence in Ghanaian preschool children. J Nutr Metab.

[3] Babatunde RO, Olagunju FI, Fakayode SB, Sola-Ojo FE (2011). Prevalence and determinants of malnutrition among under-five children of farming households in Kwara State, Nigeria. J Agric Sci.

[4] Duru CB, Oluoha UR, Uwakwe KA, Diwe KC, Merenu IA, Chigozie IO, Iwu AC (2015). Preva-lence and sociodemographic determinants of malnutrition among under-five children in rural communities in Imo state, Nigeria. Am J Public Health Res.

[5] Khan S, Zaheer S, Safdar NF (2019). Determinants of stunting, underweight and wasting among children *<* 5 years of age: evidence from 2012-2013 Pakistan demographic and health survey. BMC Public Health.

[6] Gupta AK, Borkotoky K (2016). Exploring the multidimensional nature of anthropometric indicators for under-five children in India. Indian J Public Health.

[7] Kassie GW, Workie DL (2019). Exploring the association of anthropometric indicators for underfive children in Ethiopia. BMC Public Health.

[8] Ajao KO, Ojofeitimi EO, Adebayo AA, Fatusi AO, Afolabi OT. Influence of family size, household food security status, and child care practices on the nutritional status of underfive children in Ile-Ife, Nigeria. Afr J Reprod Health. 2010;14(4):1-10. 21812205

[9] Kang Y, Aguayo VM, Campbell RK, West KP (2018). Association between stunting and early childhood development among children aged 3659 months in South Asia. Matern Child Nutr.

[10] Wolde M, Berhan Y, Chala A (2015). Determinants of underweight, stunting and wasting among schoolchildren. BMC Public Health.

[11] Akombi BJ, Agho KE, Merom D, Hall JJ, Renzaho AM (2017). Multilevel analysis of factors associated with wasting and underweight among children under-five years in Nigeria. Nutrients..

[12] Mawa R, Lawoko S (2018). Malnutrition among children under five years in Uganda. Am J Health Res.

[13] Lema K, Murugan R, Tachbele E (2018). Prevalence and associated factors of pneumonia among under-five children at public hospitals in Jimma zone, South West of Ethiopia, 2018. J Pulmonol Clin Res.

[14] Briend A, Khara T, Dolan C (2015). Wasting and stuntingsimilarities and differences: policy and programmatic implications. Food Nutr Bull.

[15] NDHS (2018). Nigerian Demographic and Health Survey.

[16] Islam MA, Mamun ASMA, Hossain MM, Bharati P, Saw A, Lestrel PE, Hossain MG (2019). Prevalence and factors associated with early initiation of breastfeeding among Bangladeshi mothers: a nationwide cross-sectional study. PloS one.

[17] Tekile AK, Woya AA, Basha GW (2019). Prevalence of malnutrition and associated factors among under-five children in Ethiopia: evidence from the 2016 Ethiopia demographic and health survey. BMC Res Notes.

[18] Agresti A (2018). An introduction to categorical data analysis.

[19] Gauvreau K, Pagano M (1997). The analysis of correlated binary outcomes using multivariate logistic regression. Biom J.

[20] Thomas Y. Fitting vector generalized linear and additive models using VGAM Package of R. Retrieved April 2020.

[21] Kassie GW, Workie DL (2020). Determinants of under-nutrition among children under five years of age in Ethiopia. BMC Public Health.

